# Video colposcopy versus headlight for large loop excision of the transformation zone (LLETZ): a randomised trial

**DOI:** 10.1007/s00404-021-06331-0

**Published:** 2021-11-21

**Authors:** Günther A. Rezniczek, Nadja Neghabian, Sadia Rehman, Clemens B. Tempfer

**Affiliations:** Department of Obstetrics and Gynecology, Marien Hospital Herne, Klinikum der Ruhr-Universität Bochum, Hölkeskampring 40, 44625 Herne, Germany

**Keywords:** Cervical dysplasia, Video colposcopy, Conisation, LLETZ, LEEP, Headlight

## Abstract

**Purpose:**

To compare resected cone mass and resection margin status when performing Large Loop Excision of the Transformation Zone (LLETZ) using video colposcopy (LLETZ-VC) versus a headlight (LLETZ-HL) in women with cervical dysplasia.

**Methods:**

Prospective, randomised trial (monocentric) at a specialised cervical dysplasia unit in a University Hospital. Women with a biopsy-proven CIN2 + or persisting CIN1 or diagnostic LLETZ were recruited and randomised. LLETZ was performed either under video colposcopic vision or using a standard surgical headlight. The primary endpoint was resected cone mass. Secondary endpoints were the rate of involved margins, fragmentation of the specimen, procedure time, time to complete haemostasis (TCH), blood loss, pain, intra- and postoperative complications, and surgeon preference.

**Results:**

LLETZ-VC and LLETZ-HL (109 women each) had comparable cone masses (1.57 [0.98–2.37] vs. 1.67 [1.15–2.46] grams; *P* = 0.454). TCH was significantly shorter in the LLETZ-VC arm (60 [41–95.2] vs. 90 [47.2–130.2] seconds; *P* = 0.008). There was no statistically significant difference in involved resection margins (6/87 [6.5%] vs. 16/101 [13.7%], *P* = 0.068) and postoperative complications (13/82 [13.7%] vs. 22/72 [23.4%], *P* = 0.085). Patient-reported outcomes favoured LLETZ-VC with a lower use of analgesics (6/80 [7.0%] vs. 17/87 [16.3%]; *P* = 0.049). However, LLETZ-VC was more difficult to perform with significantly lower ratings for handling (7 [5–9] vs. 9 [8–10]; *P* < 0.001) and general satisfaction (7.5 [5–9] vs. 10 [8–10]; *P* < 0.001).

**Conclusion:**

Intraoperative video colposcopy for LLETZ has minimal benefits at the cost of surgeons’ satisfaction.

**Clinical trial registration:**

NCT04326049 (ClinicalTrials.gov).

## Introduction

Cervical Intraepithelial Neoplasia (CIN) is a major health concern affecting women worldwide. In the United States, for example, an estimated 216 000 CIN2 + cases were diagnosed in 2016 [1]. If left untreated, high-grade cervical dysplasia such as CIN2 + may progress to cervical cancer in 40–85% of cases depending on Human Papilloma Virus (HPV) subtype infection, host immunocompetence, and associated factors of progression such as smoking and concomitant infections [2]. The standard of care for the treatment of CIN2 + is Large Loop Excision of the Transformation Zone (LLETZ) which has replaced traditional cold-knife conization of the cervix [3]. In a meta-analysis of 26 randomized trials, the incidence of persistent or recurrent CIN after LLETZ was comparable with that after cold-knife conization, but LLETZ was faster, caused less intraoperative bleeding, and resulted in a shorter hospital stay. Furthermore, LLETZ cones had overall less volume and weight and resulted in less cervical stenosis and fewer unsatisfactory examinations [4]. The use of LLETZ as the preferred surgical technique for treating cervical dysplasia is, therefore, well established, but issues remain as to the optimal adjuncts for performing LLETZ. For example, LLETZ may be performed under colposcopic guidance using a video colposcope to guarantee a sharp and magnified image. This may have advantages regarding the visibility of the cervix, the cervical canal, and cervical lesions as well as the identification of appropriate resection limits [5]. Exact visibility and high magnification may also allow for an easier and quicker coagulation of bleeding spots. Therefore, video colposcopy has the potential to provide better surgical results, especially when performed by trainees or surgeons unfamiliar with a binocular colposcope. Despite these potential advantages, the use of video colposcopy during LLETZ is not generally accepted. While video colposcopy is typically used in dysplasia outpatient clinics for the assessment of the cervix, judgement of acetowhite lesions, PAP screening, and colposcopically directed biopsies [6, 7], the usefulness of video colposcopy in the context of surgery is less clear. Specifically, to date, there is no evidence from randomised trials demonstrating benefits of video colposcopy during LLETZ. In a PubMed literature search, for example, we identified no randomised trial on video colposcopy (search date 04-04-2021; search terms: (“LLETZ”[All Fields] OR “LEEP”[All Fields]) AND (“intraop”[All Fields] OR “intraoperative”[All Fields] OR “intraoperatively”[All Fields]) AND (“colposcopy”[MeSH Terms] OR “colposcopy”[All Fields] OR “colposcopies”[All Fields])). In view of this limited body of evidence and the potential benefits of video colposcopy, we performed an adequately powered randomised trial to answer this clinically relevant issue. Assuming that video colposcopy leads to a more accurate surgical performance, we chose resected cone mass as the primary endpoint for this trial.

## Methods

Women referred to the dysplasia outpatient clinic of the Department of Obstetrics and Gynaecology, Ruhr-Universität Bochum, Bochum, Germany, were recruited for this prospective, randomised (1:1 ratio) study. Study approval was obtained by the local ethics board (Registration Number 19-6619). In addition, this trial was registered at the ClinicalTrials.gov website (registration number NCT04326049). Women were eligible if they were scheduled for LLETZ based on the following inclusion criteria: written informed consent, presence of a biopsy-proven CIN2 + or persisting CIN1 or diagnostic LLETZ in case of an abnormal Pap smear result and a type T3 transformation zone and/or an unsatisfactory colposcopy. Prior to LLETZ the number and extent of cervical lesions was assessed by colposcopy in all women. Exclusion criteria were as follows: pregnancy, a personal history of cervical surgery, presence of a concomitant oncological or haematologic disorder, and the use of blood thinners.

LLETZ was performed under local anesthesia or under general anesthesia (depending on patient preference) in an outpatient setting with same day discharge. Vasoconstrictive agents were not used. Randomisation was performed using a computer-generated list with a block size of two and the study group allocations sealed in sequentially numbered opaque envelopes. Generation of the list and sealing of the envelopes was performed by staff not involved in patient treatment. Patients were blinded to the study group allocation. The envelopes were opened by support staff in the operating room before the start of LLETZ. In women assigned to group 1 (LLETZ-VC), LLETZ was carried out with a video colposcope (Vidan^®^ 2 full-HD Video Colposcope, Schmitz, Germany) using a × 30 magnification. First, the cervix was visualised and 5% acetic acid was applied for 1 min [8]. Then, an appropriately sized electrical loop was chosen and the electrosurgical unit (Vio 300D; Erbe, Tübingen, Germany) was set at 120 watts, blend 3, high-cut mode (effect 4, 180 watts). LLETZ was carried out and the transformation zone was removed with one pass through the cervical tissue. An endocervical portion (“cowboy hat” / “sombrero”) or endocervical curettage were not performed. The resection of additional ectocervical tissue was allowed if the acetowhite lesion was not fully excised. Spray or forced coagulation modes were used to achieve appropriate haemostasis. Sutures were not used. During all procedural steps, the video colposcope was used. In women assigned to group 2 (LLETZ-HL), surgeons performed the same procedure with the use of a standard surgical headlight (Integra DUO LED Surgical Headlight System, Integra GmbH, Ratingen, Germany) to allow for an optimal brightness within the vagina and on the cervix. The headlight was chosen as the comparator intervention to rule out that LLETZ-VC may result in a better performance based solely on improved lightening of the vagina and the cervix provided by the strong LED lights surrounding the VC camera.

The primary endpoint of the study was resected cone mass measured in grams. The removed tissue was weighed with a precision scale (Kern KB-N, Kern, Germany) located in the operating room. Cone mass was chosen as the primary endpoint, because the means to accurately weigh specimens in the operating room with a precision scale were easier to set up and the measurement process less demanding than other methods for volume determination such as submersion volumetry. The volume of the cone was approximated using the pyramid formula (length × width × depth ÷ 3) based on measurements of length, width, and depth of the excised cone with a ruler immediately after surgery and before the tissue was put into formaldehyde. The pathologist report provided these measurements as well. Resection margin status (RMS) of the surgical specimen was judged according to the histopathological report. Pathologists were unaware of the study assignment. Intraoperative blood loss was measured using the difference in haemoglobin measured on the day before LLETZ and 4–5 h after LLETZ. Procedure time and time to complete haemostasis (TCH) were accurately determined in the operating room using a stopwatch following the surgeon’s commands “start” and “stop”. Additional secondary endpoints were cone fragmentation and the number of additional resections as well as intraoperative and postoperative complications (e.g. prolonged bleeding, unscheduled re-admission, cervical, uterine, or urinary tract infection) assessed by telephone interview 14 days after LLETZ. All surgeons rated the procedure with an 11-step scale ranging from 0 (worst) to 10 (best) and were free to add written comments on the two LLETZ techniques.

Study data were collected and managed using REDCap [9]. For analyses, continuous data were compared by Student’s t test or the Mann–Whitney *U* test (depending on whether the data followed a normal distribution or not, respectively) and frequencies were compared by the chi square test or Fisher’s exact test. All *P* values are two-tailed and a value < 0.05 was considered statistically significant. Values are given as means ± standard deviations, medians (interquartile ranges), or absolute numbers (percentage proportions), as appropriate. For multivariate linear and logistic regression analyses, we used total resected mass, involved margin status, and cone fragmentation as the dependent variables and age, body mass index, parity, type of transformation zone, smoking, degree of cervical dysplasia, and performed technique (LLETZ-VC or LLETZ-HL) as independent variables. Learning phase effects were evaluated comparing the pooled data of the first three procedures of every surgeon with all subsequent procedures. We used the statistics software package SigmaPlot 14.5 (Systat Software Inc., San Jose, CA).

The sample size was calculated based on the study hypothesis that LLETZ-VC would produce significantly smaller cone specimens. The assumption of a reduction of 30% of the cone mass in women undergoing LLETZ-VC was based on a previous non-randomised case–control study demonstrating a mean reduction in volume of 30% by intraoperative binocular colposcopy [10]. Based on our own previous results, we assumed a LLETZ cone mass of (2.5 ± 1.6) g (mean ± standard deviation), with the data not following a normal distribution (median of 2.1 g) [11]. Thus, with an assumed *α* of 0.05 (type I error) and an assumed drop-out rate of 5%, 88 patients (total of 176; 1:1 randomization, one-tailed) needed to be recruited for each group to achieve a power of at least 90% to confirm the superiority of LLETZ-VC regarding the primary endpoint. We used G*Power 3.1.9.2 to perform these calculations [12].

## Results

**Fig. 1:**
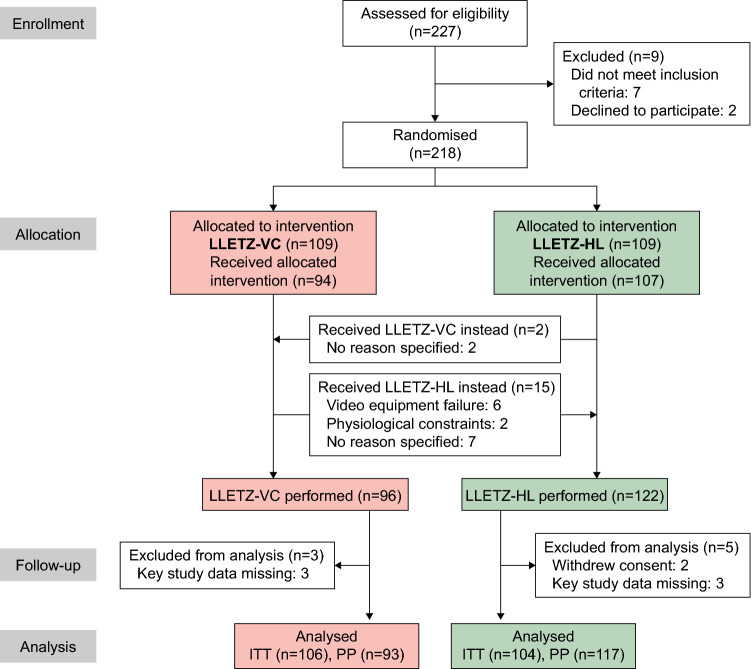
CONSORT flowchart

Between April 2020 and March 2021, we assessed 227 patients for eligibility and randomised 218 women, 109 into the LLETZ-VC arm and 109 into the LLETZ-HL arm. Figure 1 shows the flow of patients through the study. Eight patients had to be eliminated from the analysis (three from the LLETZ-VC arm and five from the LLETZ-HL arm) due to insufficient data (six cases) or withdrawal of consent after randomisation (two cases). Patient characteristics of the study participants, colposcopy results obtained prior to LLETZ (including cytological smear data and type of transformation zone), and histopathological results after LLETZ are shown in Table 1. The patient population was well balanced for all items.

**Table 1:** Patient characteristics, cytology, colposcopy, and histopathology

	Group 1: LLETZ-VC	Group 2: LLETZ-HL
Number of patients	106 (50.5)	104 (49.5)
Patient characteristics		
Age, y	37.6 (31.3–45.4)	37.1 (31.7–45.5)
Body mass index, kg/m^2^	23.4 (21.5–27.1) [1]	23.3 (21.0–26.2)
Parity	1 (0–2) [2]	1 (0–2)
Allergies (yes/no)	41 (39.0)/64 [1]	32 (31.1)/71 [1]
Tobacco use	[1]	
Currently smoking	45 (42.9)	42 (40.4)
Ever smoked	51 (48.6)	52 (50.0)
Never smoked	54 (51.4)	52 (50.0)
Regular alcohol consumption (yes/no)	3 (2.9)/102 [1]	4 (3.8)/100
Drug abuse (yes/no)	5 (4.8)/100 [1]	4 (3.8)/99 [1]
Concomitant disease (yes/no)	48 (45.7)/57 [1]	49 (47.1)/55
Prescription drug use (yes/no)	61 (58.1)/44 [1]	60 (57.7)/44
Immunosuppressive conditions (yes/no)	7 (6.7)/98 [1]	8 (7.8)/95 [1]
HPV status (positive/negative)	71 (93.4)/5 [30*]	76 (91.6)/7 [21*]
Referring cytology		
NILM	7 (6.6)	1 (1.0)
ASC-US	8 (7.5)	5 (4.8)
AGC endocervical NOS	3 (2.8)	0
ASC-H	8 (7.5)	9 (8.7)
AGC endocervical favor neoplastic	2 (1.9)	1 (1.0)
LSIL	16 (15.1)	32 (30.8)
HSIL	60 (56.6)	53 (51.0)
AIS	1 (0.9)	3 (2.9)
HSIL with features suspicious for invasion	1 (0.9)	0
Type of transformation zone		
Type 1	64 (60.4)	61 (58.7)
Type 2	14 (13.2)	21 (20.2)
Type 3	28 (26.4)	22 (21.2)
Indication for LLETZ		
Biopsy with HSIL	53 (50.0)	49 (47.1)
Biopsy with LSIL	31 (29.2)	33 (31.7)
Abnormal Pap or inconclusive colposcopy	22 (20.8)	22 (21.2)
Histology result after LLETZ		
Negative for dysplasia	2 (1.9)	2 (1.9)
LSIL	20 (18.9)	25 (24.0)
HSIL	82 (77.4)	74 (71.2)
AIS	1 (0.9)	0
Invasive cancer	1 (0.9)	3 (2.9)

Values are counts (percentage proportions) or medians (interquartile ranges). Numbers in square brackets indicate the number of missing values

*LLETZ* large loop excision of the transformation zone, *VC* video colposcope, *HL* surgical headlight, *HPV* human papilloma virus, *AGC* atypical glandular cells, *AIS* adenocarcinoma in situ, *ASC* atypical squamous cells, *ASC-US* ASC of undetermined significance, *ASC-H* ASC cannot exclude HSIL, *HSIL* high grade squamous intraepithelial lesion, *LSIL* low grade squamous intraepithelial lesion, *NILM* negative for intraepithelial lesion or malignancy, *NOS* not otherwise specified

*Unknown HPV status

Table 2 shows the primary and secondary study endpoints (intention to treat analysis, ITT). The primary endpoint of the study, i.e. cone mass, did not differ between the study groups (1.57 [0.98–2.37] vs. 1.67 [1.15–2.46] grams; *P* = 0.454). Regarding the secondary endpoints, the groups did differ from each other. TCH was significantly shorter in the LLETZ-VC arm (60 [41–95.2] vs. 90 [47.2–130.2] seconds; *P* = 0.008). In addition, LLETZ-VC led to a lower rate of involved resection margins (6/87 [6.5%] vs. 16/101 [13.7%]) and fewer postoperative complications (13/82 [13.7%] vs. 22/72 [23.4%]), but these differences were not statistically significant (*P* = 0.068 and *P* = 0.085, respectively). On the other hand, LLETZ-VC was more difficult to perform and thus received significantly lower ratings by the study surgeons for handling of the technique (7 [5–9] vs. 9 [8–10]; *P* < 0.001) and general satisfaction (7.5 [5–9] vs. 10 [8–10]; *P* < 0.001). All other secondary endpoints, including cone dimensions, cone volume, cone fragments, and intraoperative complications, did not differ between the two study arms.

**Table 2:** Primary and secondary outcome measures

	LLETZ-VC	LLETZ-HL	*P*
Number of patients	106	104	
Primary outcome			
Cone mass, g	1.57 (0.98–2.37) [1]	1.67 (1.15–2.46) [1]	0.454
Total resected mass, g	1.84 (1.26–2.62) [1]	2.14 (1.35–2.90) [1]	0.300
Secondary outcomes			
Cone dimensions (intra-OP)	[2]		
Base length, mm	18 (15–21)	17.5 (15–21)	0.679
Base width, mm	15 (12–18.7)	15.5 (12–18)	0.661
Depth, mm	9 (6–11)	9 (6–11)	0.796
Volume,^a^ cm^3^	0.73 (0.43–1.21)	0.75 (0.46–1.11)	0.933
Cone dimensions (pathologist)			
Base length, mm	22 (18.7–25)	21 (18–25)	0.484
Base width, mm	16 (14–19)	17 (14–19)	0.467
Depth, mm	9 (7–12.2)	10 (7–12)	0.631
Volume,^a^ cm^3^	1.10 (0.60–1.67)	1.10 (0.60–1.70)	0.600
Number of fragments (> 1 vs 1)	4 (3.8) / 102	5 (4.8) / 99	0.711
Number of additional resections	1 (0–1)	1 (0–1)	0.894
Resection margin status (R1 vs R0)	7 (6.6) / 99	15 (14.4) / 89	0.064
Procedure duration, seconds	164 (112.5–234) [2]	189 (117–287) [1]	0.160
TCH, seconds	60 (41–95.2) [10]	90 (47.2–130.2) [12]	**0.008**
Intraoperative blood loss, ΔHb	0.70 ± 0.68 [19]	0.51 ± 0.71 [20]	0.077
Surgeons’ ratings	[4]	[8]	
Handling of the technique	7 (5–9)	9 (8–10)	** < 0.001**
General satisfaction	7.5 (5–9)	10 (8–10)	** < 0.001**
Complications			
Intraoperative, yes vs no	8 (7.5) / 98	8 (7.7) / 96	0.968
Postoperative, yes vs no	13 (13.7) / 82 [11]	22 (23.4) / 72 [10]	0.085

Values are medians (interquartile ranges) or counts (percentage proportions). Numbers in square brackets indicate the number of missing values. Analysis is by intention to treat. *P* values were calculated using the *χ*^2^ or Mann–Whitney *U* tests, as appropriate; statistical significance (*P* < 0.05) is highlighted in bold

*LLETZ* large loop excision of the transformation zone, *VC*video colposcope, *HL* surgical headlight, *TCH* time to complete haemostasis, *ΔHb* difference in haemoglobin levels

^a^Cone volume was calculated as length × width × height ÷ 3 (pyramid)

Two patients randomised to LLETZ-HL crossed over to LLETZ-VC and vice versa 15 patients crossed over to LLETZ-HL (Fig. 1). Therefore, we also performed a per-protocol (PP) analysis of the primary and secondary endpoints, which showed the same results as the ITT analysis with a shorter TCH in favor of LLETZ-VC (60 [40–90] vs. 90 [48–133.5] seconds; *P* < 0.001) and lower ratings for LLETZ-VC regarding handling of the technique (*P* < 0.001) and general satisfaction (*P* < 0.001). All other outcomes did not differ between groups in agreement with the ITT analysis (data not shown). Of the 35 post-operative complications observed, 11/86 (12.8%) occurred in patients treated with LLETZ-VC and 24/103 (23.3%) in patients treated with LLETZ-HL; *P* = 0.064 (per protocol analysis). In detail, complications were as follows: bleeding (*n* = 12; VC 6, HL 6), infection (*n* = 8; VC 2, HL 6), pain (*n* = 8; VC 1, HL 7), vaginal discharge (*n* = 3; VC 1, HL 2), others (*n* = 4; VC 1, HL 3).

Table 3 shows patient-reported outcomes. Pain level immediately after surgery (1 [0–3] vs. 1 [0–2]; *P* = 0.653) and overall patient satisfaction (10 [9, 10] vs. 10 [10]; *P* = 0.112) were not different between the two study arms. Fourteen days after surgery, patient satisfaction, pain level, duration of pain, bleeding severity, and bleeding duration were also not different between groups. However, patients in the LLETZ-VC arm reported a lower use of analgesics (6/80 [7.0%] vs. 17/87 [16.3%]; *P* = 0.049), but we do not consider this relevant.

**Table 3:** Patient-reported outcomes

	LLETZ-VC	LLETZ-HL	*P*
Number of patients*	93	117	
Post-procedure questionnaire			
General satisfaction	10 (9–10) [6]	10 (10–10) [3]	0.112
Current pain level	1 (0–3) [5]	1 (0–2) [2]	0.653
Follow up interview (after 14 days)			
Lost to follow-up	7 (7.5)	13 (11.1)	0.379
General satisfaction	10 (9–10) [7]	10 (10–10) [14]	0.325
Pain level (first day)	1 (0–3) [7]	0.5 (0–4) [13]	0.931
Duration of pain, days	2 (0–4) [7]	2 (0–6.75) [13]	0.863
Needed to take pain meds (yes vs. no)	6 (7.0) / 80 [7]	17 (16.3) / 87 [13]	**0.049**
Bleeding severity (first day)	1 (0–3) [8]	1 (0–3) [13]	0.589
Bleeding duration, days	3 (0–10) [8]	3 (0–10.75) [13]	0.883

Values are medians (interquartile ranges) or counts (percentage proportions). Numbers in square brackets indicate the number of missing values. *P* values were calculated using the χ^2^ or Mann–Whitney *U* tests, as appropriate; statistical significance (*P* < 0.05) is highlighted in bold

*LLETZ* large loop excision of the transformation zone, *VC* video colposcope, *HL* surgical headlight

*Number of patients are derived from the per-protocol analysis

**Fig. 2:**
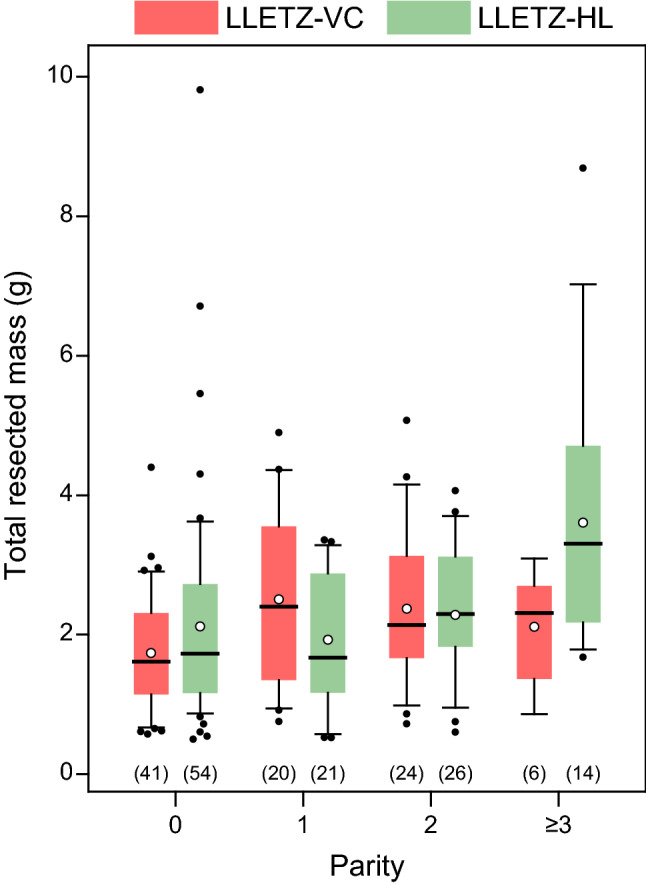
Box plots showing total resected mass broken down by parity for the two LLETZ techniques (per protocol analysis). Box plots: boundaries of the boxes, 25th/75th percentiles; thick horizontal lines, medians; whiskers, 10th and 90th percentiles; full circles, outliers; open circles, means. Number of data points are indicated in parentheses. *LLETZ* large loop excision of the transformation zone, *VC* video colposcope, *HL* surgical headlight

In a multivariate analysis, treatment group and degree of dysplasia were independent predictors of involved resection margins (*P* = 0.028 and *P* = 0.014, respectively) and—as expected—parity was an independent predictor of cone mass (*P* < 0.001) (Table 4). Of note, we observed an interaction between the two independent variables parity and treatment type. As shown in Fig. 2, the effect of LLETZ-VC leading to smaller cones was only seen in women with high parity but not in nulliparous women, where it would have the greatest clinical benefit.

**Table 4:** Multivariate analysis

Independent variables	Dependent variables						
	Resected cone mass			Involved margin status		Cone fragmentation	
	OR (CI)	*P*_*log*_	*P*_*lin*_	OR (CI)	*P*_*log*_	OR (CI)	*P*_*log*_
LLETZ-VC performed	0.90 (0.50–1.61)	0.719	0.272	0.28 (0.09–0.87)	**0.028**	1.10 (0.27–4.49)	0.899
Age	1.01 (0.97–1.04)	0.696	0.971	1.00 (0.93–1.08)	0.994	0.92 (0.82–1.04)	0.186
Body mass index	1.00 (0.94–1.06)	0.934	0.981	1.06 (0.97–1.17)	0.204	1.11 (0.99–1.24)	0.075
Parity	2.02 (1.46–2.80)	** < 0.001**	** < 0.001**	0.55 (0.29–1.03)	0.060	1.18 (0.55–2.54)	0.664
Tobacco use	0.80 (0.44–1.46)	0.473	0.371	1.45 (0.52–3.99)	0.476	0.45 (0.10–1.96)	0.286
Type of transformation zone	0.70 (0.43–1.13)	0.144	0.397	0.38 (0.13–1.18)	0.094	0.51 (0.11–2.32)	0.382
Degree of dysplasia	1.45 (0.76–2.73)	0.256	0.084	7.03 (1.48–33.33)	**0.014**	0.65 (0.15–2.85)	0.567

Multiple linear and logistic regression analyses with resected cone mass (for logistic regression, cone mass ≤ vs. > median mass of 2.00 g), involved margin status (R1 vs. R0), and cone fragmentation (yes vs. no) as dependent variables, and performed technique (1, LLETZ-VC vs. 0, LLETZ-HL), age, body mass index, parity, tobacco use (1, ever vs. 0, never), type of transformation zone (1, 2, or 3), and degree of dysplasia (0, negative; 1, low-grade squamous intraepithelial lesion; 2, high-grade squamous intraepithelial lesion; 3, carcinoma) as independent variables

*CI* confidence interval (5%–95%), *OR* odds ratio, *P*_*lin*_ multiple linear regression *P* value, *P*_*log*_ multiple logistic regression *P* value, *LLETZ* large loop excision of the transformation zone, *VC* video colposcope, *HL* surgical headlight

Lastly, we looked at learning phase effects associated with LLETZ-VC. We found that all objective variables including cone mass, cone dimensions, number of fragments, resection margin status, procedure duration, and intraoperative blood loss, as well as intra- and postoperative complications were not different between the learning and post-learning phases (data not shown). This indicates that LLETZ-VC has a steep learning curve without a measurable effect on procedural quality. It also indicates that the primary study endpoint is not affected by learning-curve effects. However, many surgeons commented on a number of difficulties related to LLETZ-VC indicating that the procedure is technically demanding.

## Discussion

LLETZ is a common surgical intervention with around 330 000 procedures performed in the US per year [13]. Therefore, improvements in the surgical performance of LLETZ are clinically relevant. Video colposcopy is a potential means of improving intraoperative visibility and surgical accuracy and is marketed as such. However, to date, there is no high-quality evidence to reliably assess the benefits of LLETZ-VC. In the present randomised trial, we demonstrated that LLETZ-VC does not result in significantly smaller cone specimens but has moderate benefits regarding a shorter coagulation time and a lower need for postoperative analgesics. Thus, our study provides high-level evidence that intraoperative video colposcopy during LLETZ may be used for convenience but has only minor health-related benefits.

LLETZ-VC provided excellent and sharp images but LLETZ-VC turned out to be technically demanding based on the surgeons’ written comments and their evaluations favoring LLETZ-HL. Therefore, we looked at learning phase effects associated with LLETZ-VC to rule out that a flat learning curve might bias study results. Clearly, this was not the case. We found that all objective variables including cone mass, cone dimensions, number of fragments, resection margin status, procedure duration, and intraoperative blood loss, as well as intra- and postoperative complications were not different between the learning and post-learning phases. This indicates that LLETZ-VC has a steep learning curve without a measurable effect on procedural quality. It also indicates that the primary study endpoint is not affected by learning-curve effects. However, surgeons’ comments included a number of difficulties related to LLETZ-VC, indicating that the procedure is technically demanding.

Video colposcopy is being used in many countries both for routine colposcopy and for cervical surgery such as LLETZ and is endorsed by many gynaecologic societies. For example, the Italian Society for Colposcopy and Cervico-Vaginal Pathology (SICPCV) explicitly recommends the use of a video colposcope both for colposcopy and outpatient surgery in the lower genital tract, especially in times of the COVID-19 pandemic [14]. In accordance, the Guideline for Cervical Cancer Screening and Treatment of Cervical Dysplasia of the German Society of Gynaecology and Obstetrics (DGGG) recommends to perform LLETZ under colposcopic guidance using either a binocular colposcope or a video colposcope [15]. However, there is only limited evidence available in the literature assessing the pros and cons of video colposcopy including a lack of randomised trials. Video colposcopy is expensive and is being marketed as a tool to improve surgical quality [16]. Therefore, our study should be helpful for clinicians to correctly assess the potential benefits or lack thereof associated with a significant financial investment in video colposcopy equipment.

It is of note that surgeons’ ratings favoured LLETZ-HL over LLETZ-VC. This differential assessment was not limited to the learning phase but persisted throughout the study. On the other hand, individual surgeons’ ratings strongly differed between consecutive patients. This suggest that LLETZ-VC may be more difficult to handle and that the degree of difficulty depends on patient characteristics such as vaginal length and tightness and cervical size.

Our study has strengths and limitations. First, the internal validity of the study is adequate with a high number of patients and a randomised study design providing a high degree of reliability of the study results. The monocentric study design assures conformity of surgical procedures. Regarding limitations, the external validity of our study may be restricted to the particular video colposcope used in the study. Other video colposcopes may provide better results. In addition, selection bias may have influenced the study results, because we only treated women referred to our specialised colposcopy center in the setting of a University Hospital. This may lead to a patient collective with selected and more severe cases and results may differ from other patient collectives, for example patients in a gynaecologic practice.

## Conclusion

In summary, we found that intraoperative video colposcopy during LLETZ does not lead to significantly smaller cone specimens but has moderate benefits regarding a shorter coagulation time and potentially a lower need for postoperative analgesics. We conclude that intraoperative video colposcopy during conisation may be used for convenience but has only minor health-related benefits.

## Data Availability

Data will be shared upon reasonable request made to the corresponding author. This includes individual participant data underlying the results presented here, after deidentification, as well as data dictionaries and the study protocol. Data are available after publication, without a specific end date. Requesting investigators must show that their proposed use of the data has been approved by an independent review committee identified for this purpose.
